# Supplementary material for: Laser-Induced Morphological and Structural Changes of Cesium Lead Bromide Nanocrystals
Source: Nanomaterials (Basel). 2022 Feb 20;12(4):703. doi: 10.3390/nano12040703 (PMC8879588; doi:10.3390/nano12040703)
Supplement: Supplementary file 1 [file nanomaterials-12-00703-s001.zip › nanomaterials-1604321-supplementary.pdf]

-Supporting Information-

Laser-induced Morphological and Structural Changes of Cesium Lead Bromide Nanocrystals

Athanasia Kostopoulou,[a,‡,*] Konstantinos Brintakis,[a,‡,*] Maria Sygletou,[a] Kyriaki Savva,[a] Nikolaos Livakas,[a,b] Michaila Akathi Pantelaiou,[a,b] Zhiya Dang,[c] Alexandros Lappas,[a] Liberato Manna,[c] Emmanuel Stratakis[a,e,*]

‡Equal contribution

*corresponding authors

akosto@iesl.forth.gr

kbrin@iesl.forth.gr

stratak@iesl.forth.gr

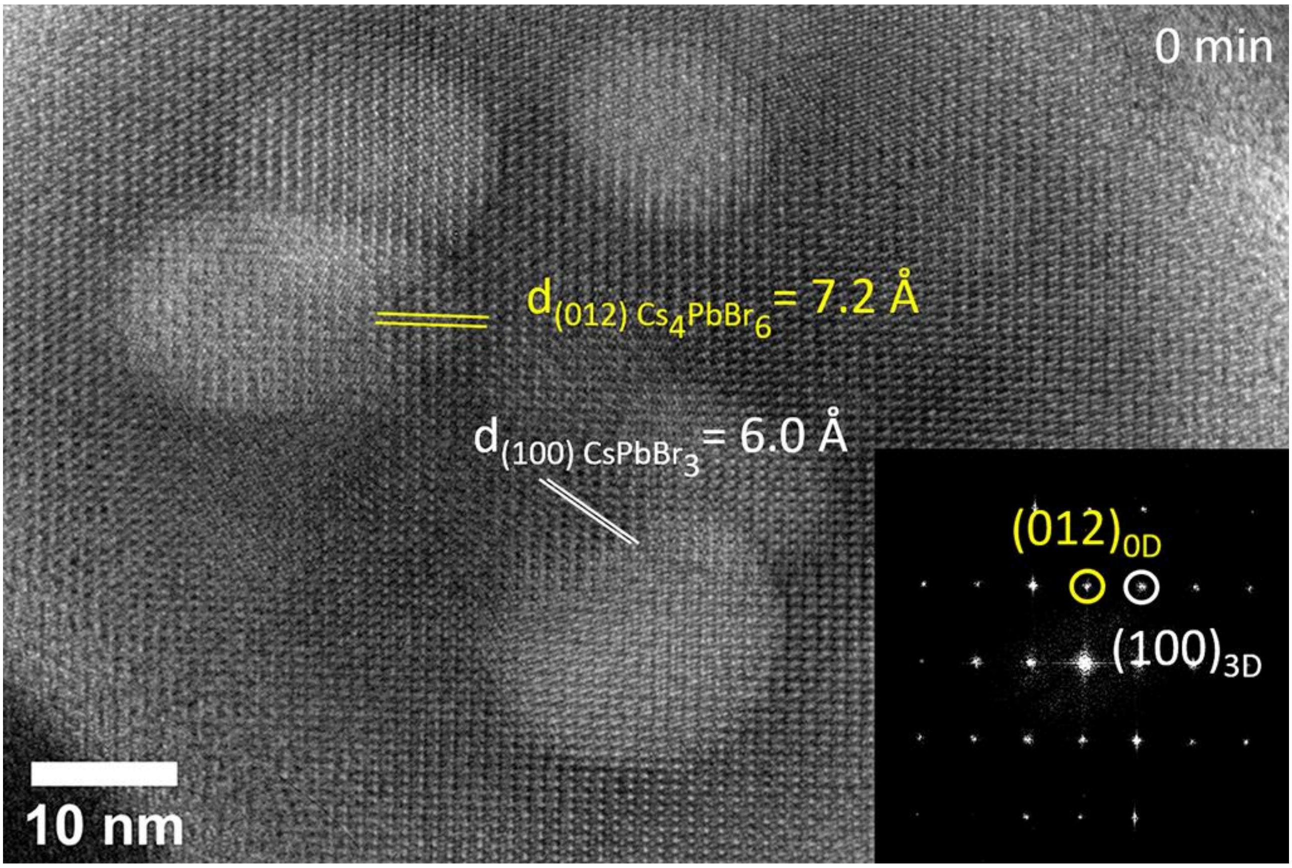

Figure S1. HRTEM image and corresponding FFT pattern of the pristine nanohexagons dispersed in DCB.

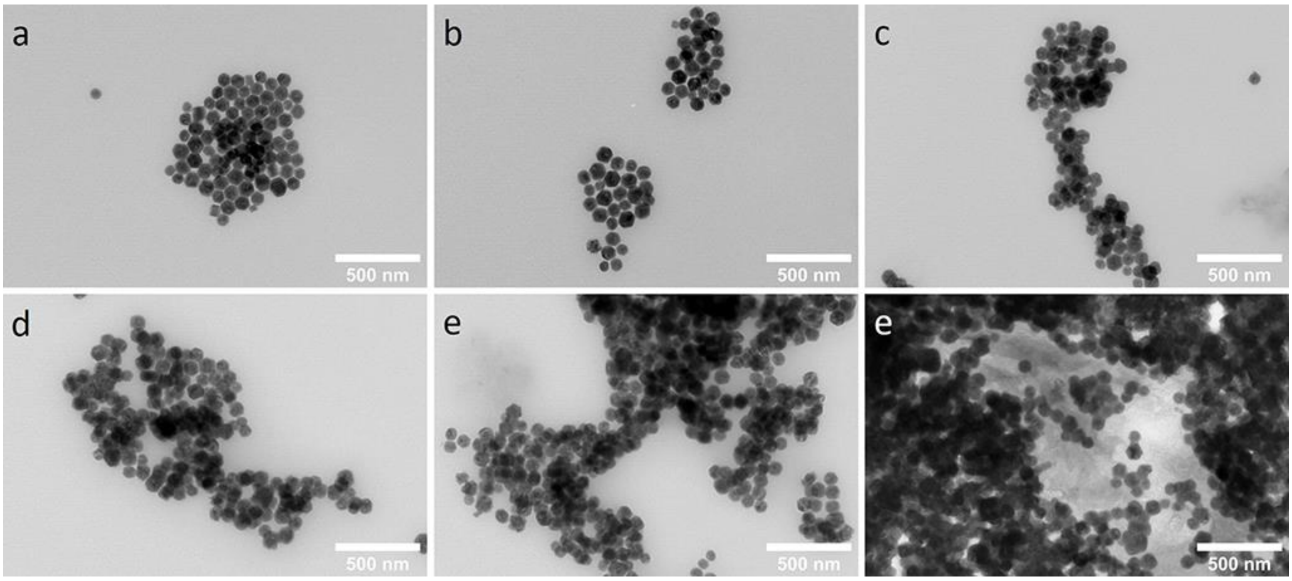

Figure S2. Low magnification TEM images of the irradiated nanocrystals with a laser fluence of 0.5 mJ/cm^2 and 100 (a), 10^3 (b), 10^4 (c), 1.8×10^6 (d), 28.8×10^6 and 57.6×10^6 (e) number of pulses.

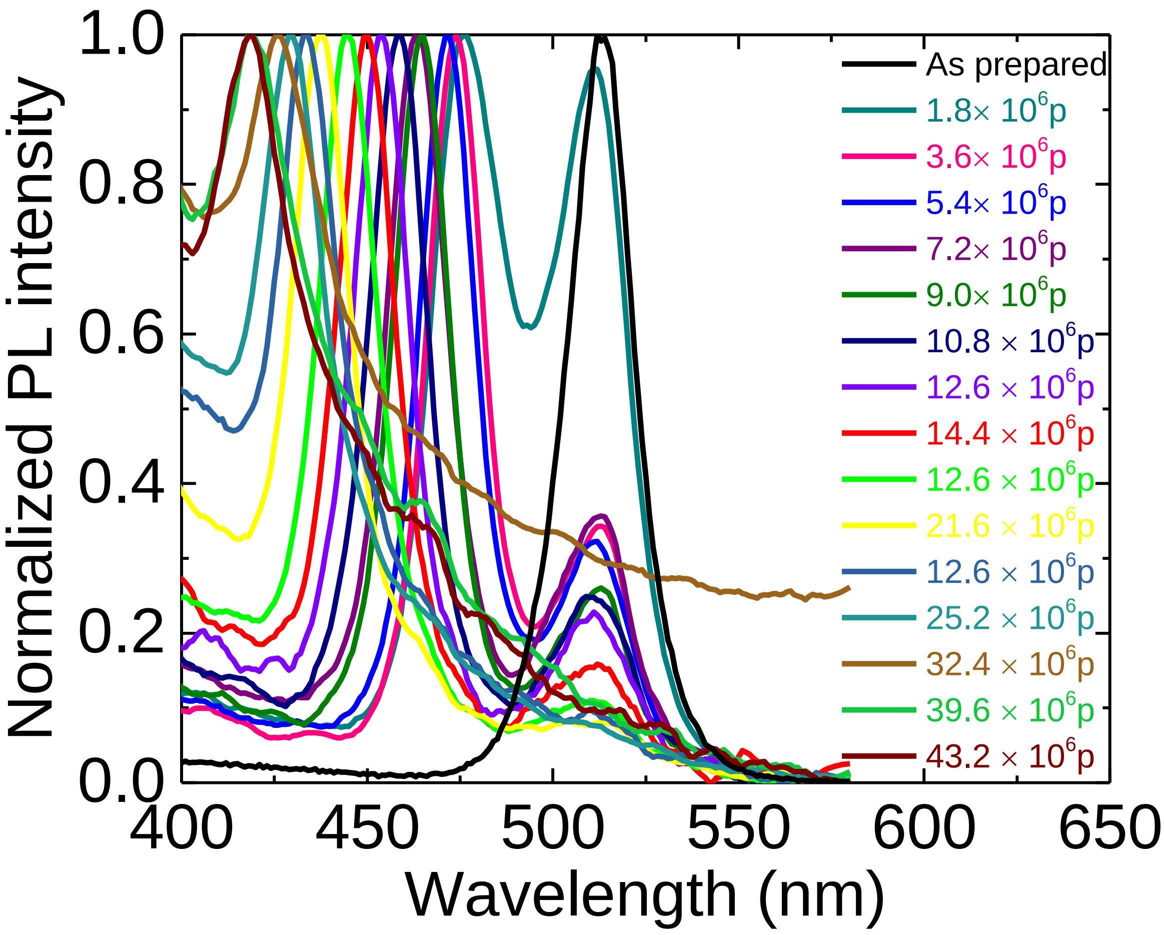

Figure S3. PL spectra of the DCB-based nanohexagons solutions irradiated with a laser fluence of 129 mJ/cm^2 and number of pulses from 1.8 to 43.6 × 10^6 pulses.

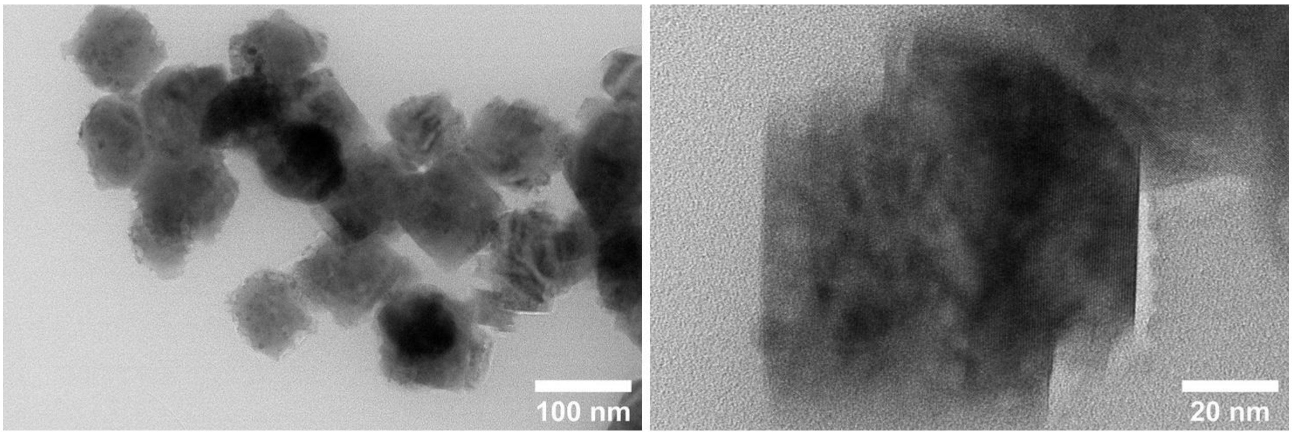

Figure S4. Exfoliated nanohexagons after irradiation with 1.8×10^6 pulses.

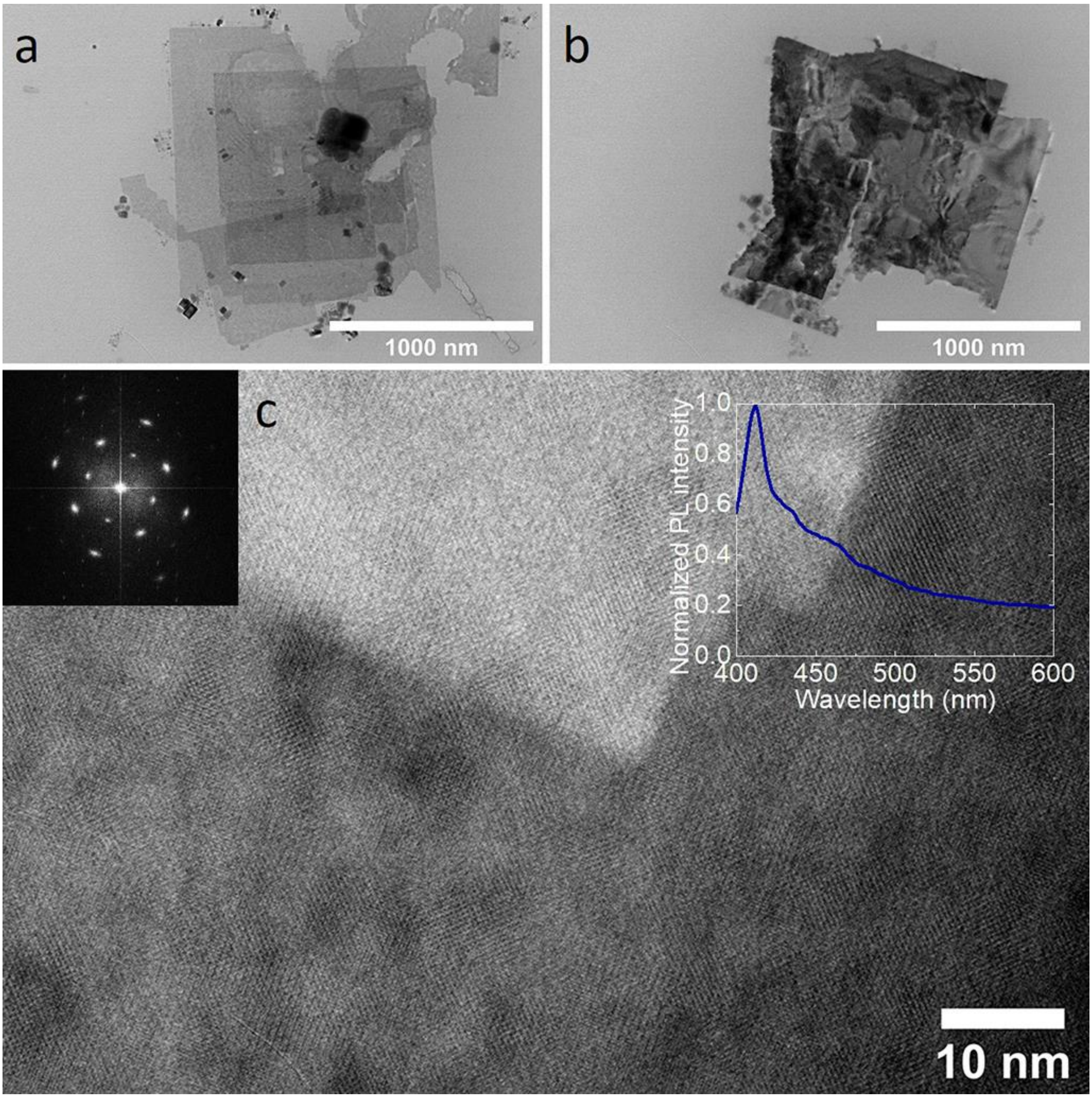

Figure S5. Low magnification (a, b) and HRTEM (c) images of the microsheets formed after irradiation with 61.2 × 10^6 pulses of the DCB-based nanohexagon solution. Insets in part c: FFT pattern of the HRTEM image (left) and PL spectrum of the same solution (right).

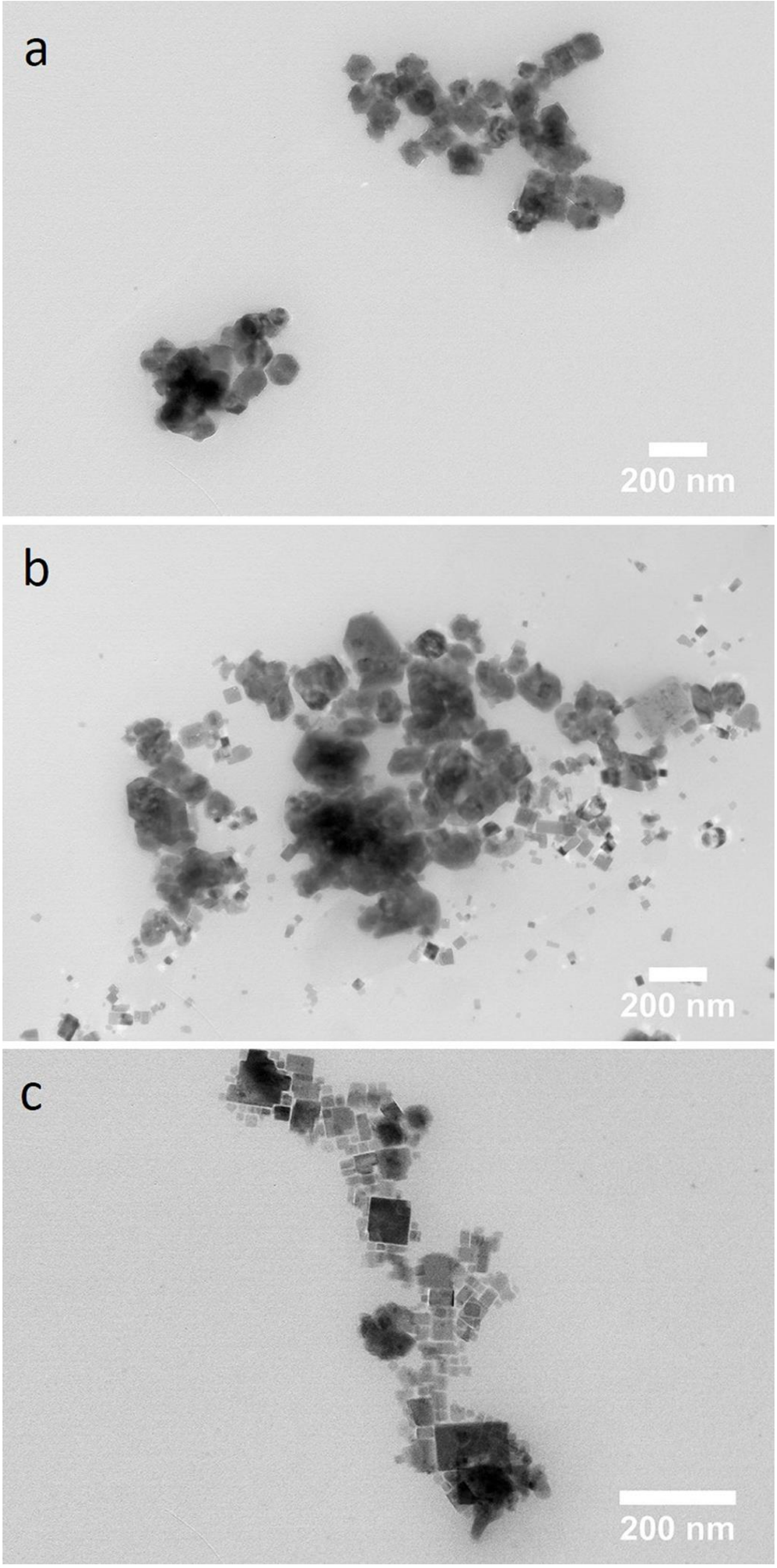

Figure S6. Low magnification TEM images of the nanocrystals irradiated with 129 mJ/cm^2 fluence and 1.8 (a), 7.2 (b) and 14.4 (c) × 10^6 pulses.

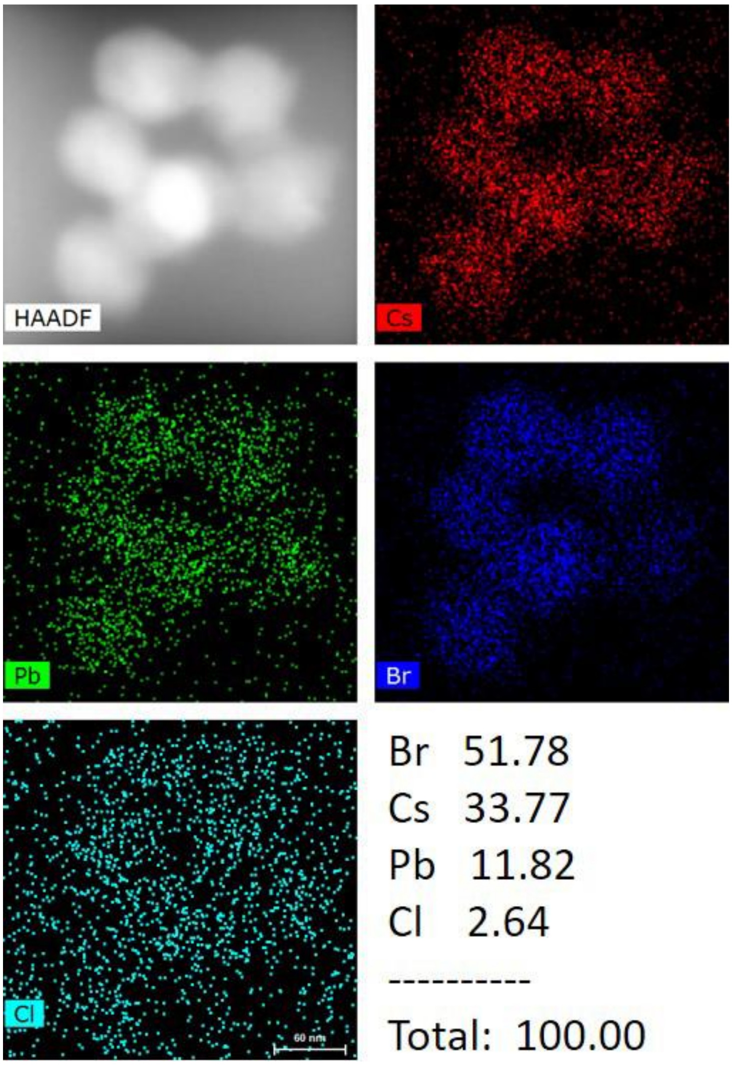

Figure S7. EDS mapping of the irradiated sample after 0.6 × 10^6 irradiation pulses and their corresponding atomic %.

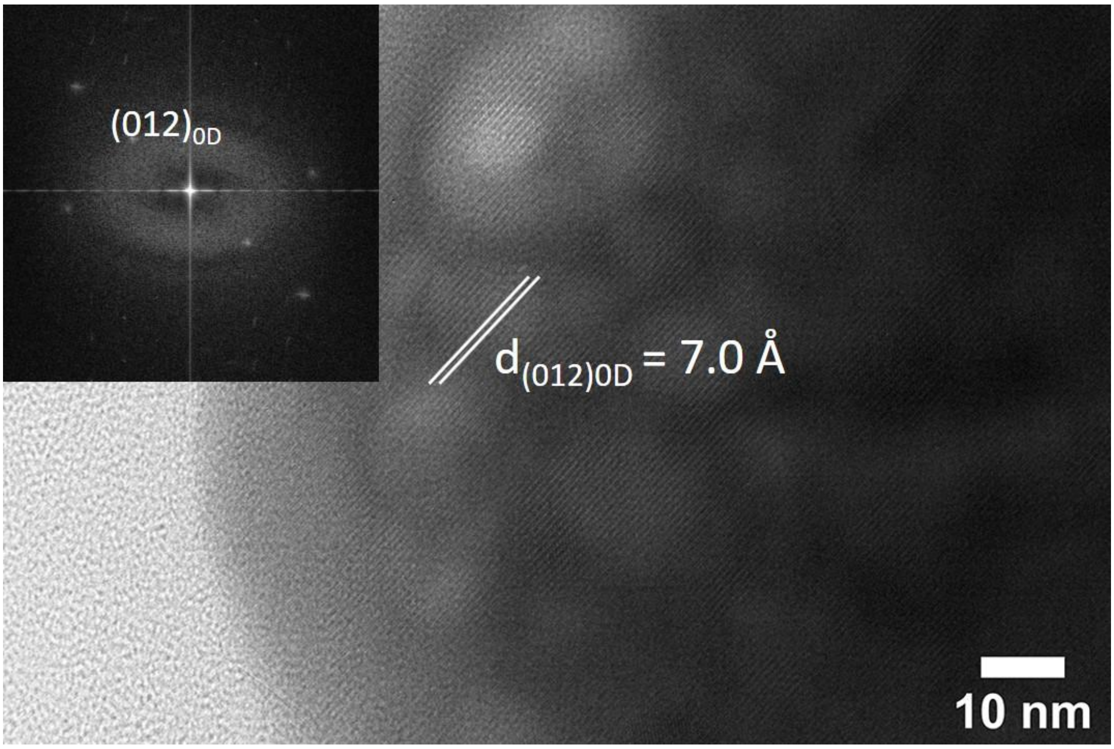

Figure S8. HRTEM image and FFT pattern of the irradiated nanohexagons with 7.2 × 10^6 pulses.

S1.1 Optimum fluence for well-shaped nanocrystals

In order to obtain the optimum results and well-formed nanocrystals, different fluences have been tested before to choose the 129 mJ/cm^2 value. Irradiation with a smaller (92 mJ/cm^2), and a larger fluence (165 mJ/cm^2) have been tested and the selection was done according to the quality of the cryctals from TEM images and their PL properties. PL spectra indicated that the anion exchange together with the morphology alteration were occurred faster for irradiation with the two larger fluences (two peaks in the black curves) (Figure S9). For 6 minutes irradiation (21.6× 10^6 pulses), the initial PL peak centered at 515 nm for non-irradiated nanohexagons was blue shifted to 461 nm, 437 nm and 428 nm for the fluences 92 mJ/cm^2, 129 mJ/cm^2 and 165 mJ/cm^2 respectively (Figure S10). In addition to the anion exchange which takes place in all the irradiation conditions, TEM images indicated also differences in the morphology of the nanocrystals (Figure S9d-i). The nanocrystals of lower and largest fluences are not well-shaped, especially in the latter case but similar in size (Figure S11).

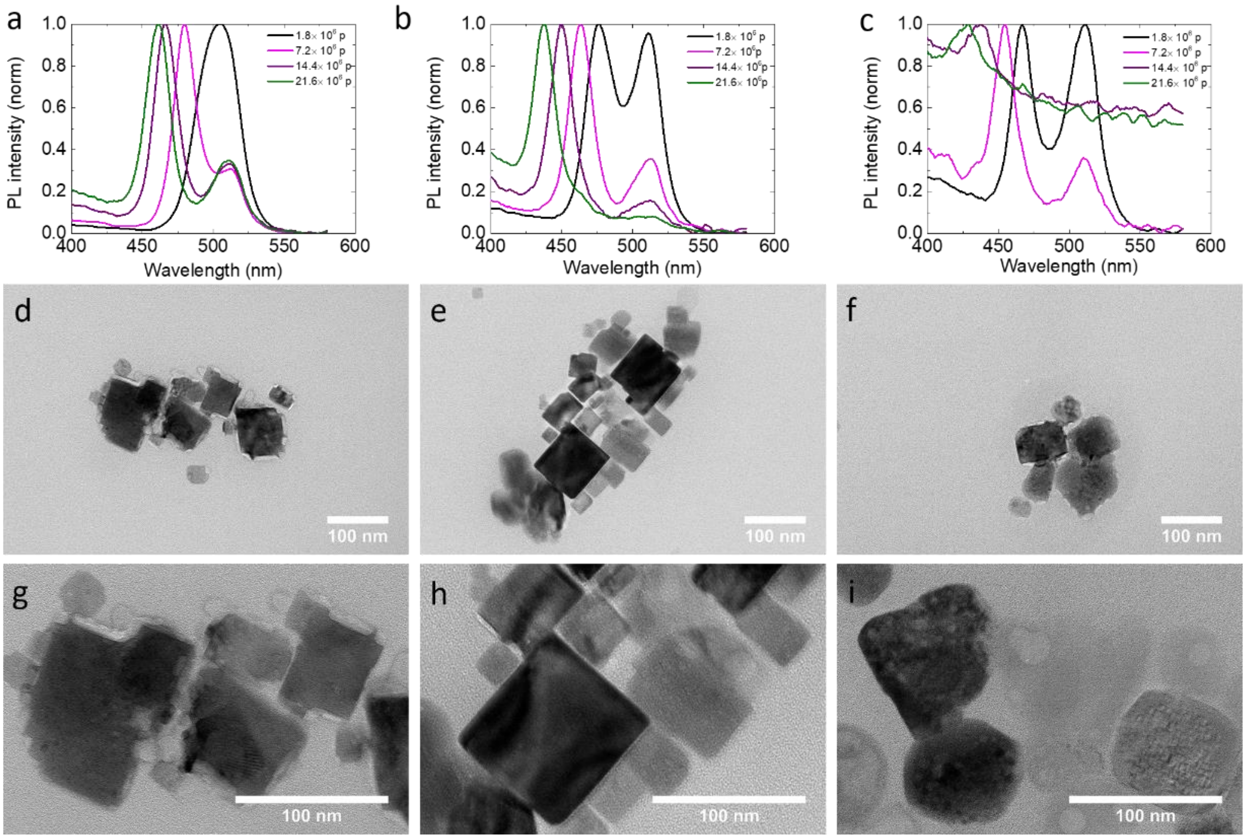

Figure S9. Photoluminescence of the DCB-based nanohexagon solutions irradiated with 92 (a), 129 (b) and 165 (c) mJ/cm^2 fluence with 1.8, 7.2, 14.4 and 21.6 × 10^6 pulses. TEM images for the same fluences after 21.6 million pulses (6 min). The irradiation carried out with a femtosecond laser of 513 nm wavelength.

The nanoplatelets after 6 min irradiation and fluence of 129 mJ/cm^2 were well-formed with sharp edges and well-crystalline (Figure 3b) while small section were removed from the nanoplatelets formed with the smaller and larger fluences (Figure 3a, c) and they were completely degraded for the higher irradiation conditions (Figure 3c). In the case of the smaller fluence, the irradiation energy is efficient for the destabilization of the surface ligands and the

fusion of the nanocubes to plateletes, but not enough to recrystalline and form well-shaped crystals. In the case of the largest fluence the energy is strong enough to degrade again the platelets. The intermediate fluence of 129 mJ/cm^2 is the optimum condition for well-formed nanoplatelets and also for morphological and structural modifications.

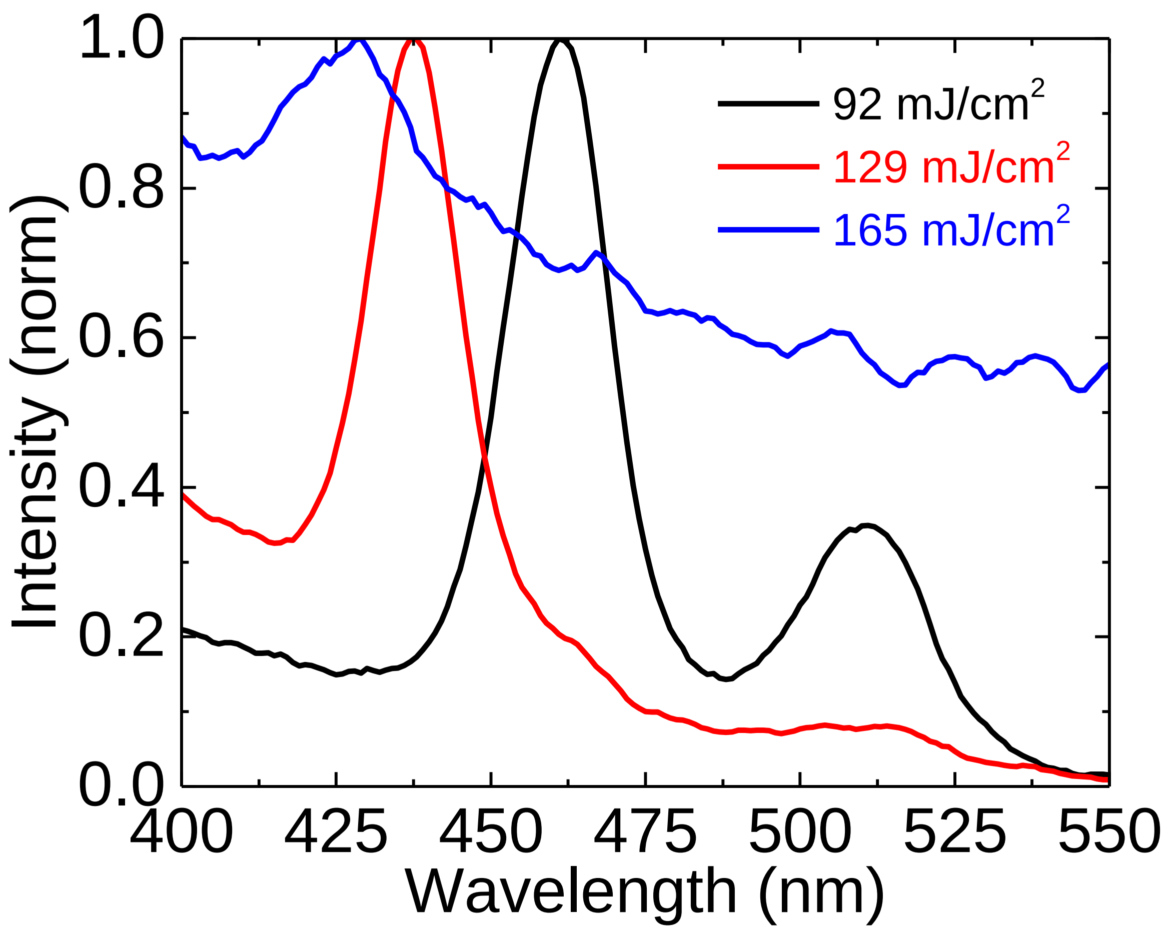

Figure S10. Photoluminescence spectra of the DCB-based nanohexagons solutions irradiated with the fluence of 92 mJ/cm^2 (black curve), 129 mJ/cm^2 (red curve) and 165 mJ/cm^2 (blue) after 21.6 million pulses (6 min) irradiation.

S1.2 Irradiation with 1026 nm laser wavelength

To study the laser wavelength dependence on the final morphological and compositional features of the nanohexagons, the DCB dispersed nanohexagons colloid was irradiated with a femtosecond laser of 1026 nm wavelength and fluence the optimum selected in the section §S1.1 for irradiation with the 513 nm laser. Besides the morphological transformation occurred upon irradiation with a 513 nm and laser fluence of 129 mJ/cm^2, the irradiation with IR wavelength same fluence seemed to do not affect the morphology of the primary nanohexagons possibly due to the poor low energy absorption from the nanocrystals at these wavelengths (Figure S12a, b). Despite the nanohexagons retained their morphologies even after long time irradiation (54×10^6 pulses, 15 min), their optical properties were changed with the PL intensity to be increased with the number of the irradiation pulses (Figure

S12c). PL enhancement has been referred to date for single crystals of metal halide perovskites upon irradiation, while the PL properties seemed not to be affected for nanocrystals of the same crystal structure.[1,2] Particularly, PL intensity enhancement has been observed by femtosecond laser processing of single crystal organic-inorganic halides by introducing micro-/nanostructures on their surface[2], while no change of the PL properties observed for $CsPbBr_3$ nanocubes upon three hours laser irradiation with laser wavelength of 1064 nm[1].

Low magnification TEM images indicated that no fragmentation was occurred under irradiation with these conditions (Figure S11a), even after 15 min. that could be an explanation for such PL enhancement[1]. In addition, the PL intensity enhancement could be attributed to phase transformation to a PL active phase. Phase transformations have been observed by laser irradiation of metal halide perovskite materials with moderate-to-high fluence (greater than 0.34 mJ/cm^2). $CsPbBr_3$ nanocrystals were experienced significant impulsive heating causing a reversible, photoinduced orthorhombic-to-cubic phase transition.[3] In addition, phase transformation from orthorhombic to tetragonal phase has been occurred in $CsPbBr_3$ single crystals with above-bandgap illumination (1.6 mW cm^{-2}, 532 nm laser wavelength)[3]. In our case, the non-existence of individual nanocubes in TEM images which would indicate the fragmentation and phase transformation from the Cs_4PbBr_6 to the $CsPbBr_3$ phase similar to that observed upon irradiation with 513 nm wavelength laser, lead to the hypothesis that the observed PL enhancement is originated mainly from phase transformation inside the Cs_4PbBr_6 phase. Increase of the $CsPbBr_3$ inclusions in the Cs_4PbBr_6 upon irradiation has been observed in XRD pattern even in lower fluence for the same number of pulses (Figure S12).

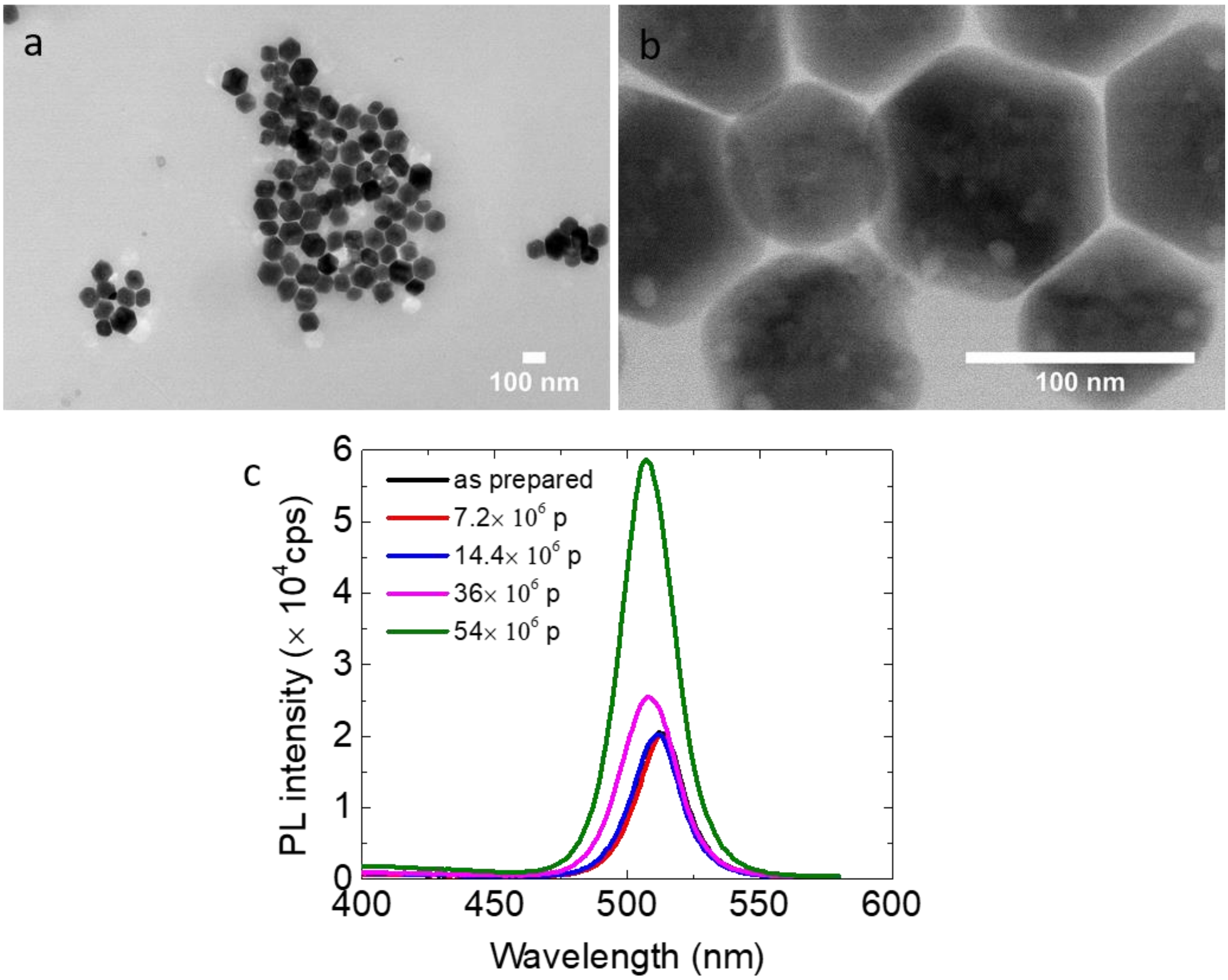

Figure S11. TEM images of the nanohexagons after 54 × 10^6 pulses (a-b) and photoluminescence spectra upon a range of pulses (c). The irradiation conducted with a femtosecond laser of 1026 nm wavelength and fluence of 129 mJ/cm^2.

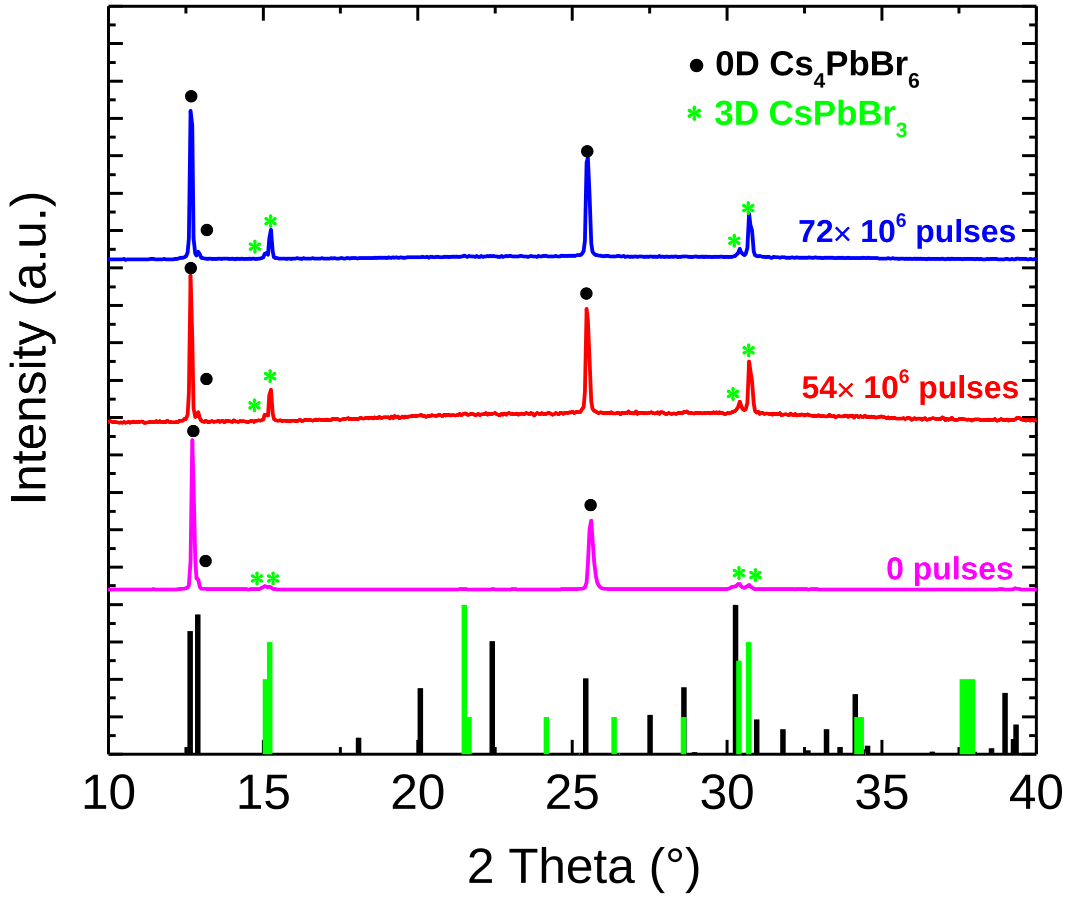

Figure S12. X-ray diffraction pattern of the nanohexagons before and after irradiation with laser fluence of 44.6 mJ/cm^2.

References

(1) Dong, Y.; Hu, H.; Xu, X.; Gu, Y.; Chueh, C.-C.; Cai, B.; Yu, D.; Shen, Y.; Zou, Y.; Zeng, H. Photon-Induced Reshaping in Perovskite Material Yields of Nanocrystals with Accurate Control of Size and Morphology. *J. Phys. Chem. Lett.* **2019**, *10* (15), 4149–4156. https://doi.org/10.1021/acs.jpclett.9b01673.

(2) Xing, J.; Zheng, X.; Yu, Z.; Lei, Y.; Hou, L.; Zou, Y.; Zhao, C.; Wang, B.; Yu, H.; Pan, D.; Zhai, Y.; Cheng, J.; Zhou, D.; Qu, S.; Yang, J.; Ganeev, R. A.; Yu, W.; Guo, C. Dramatically Enhanced Photoluminescence from Femtosecond Laser Induced Micro-/Nanostructures on MAPbBr3 Single Crystal Surface. *Adv. Opt. Mater.* **2018**, *6* (20), 1800411. https://doi.org/10.1002/adom.201800411.

(3) Kirschner, M. S.; Diroll, B. T.; Guo, P.; Harvey, S. M.; Helweh, W.; Flanders, N. C.; Brumberg, A.; Watkins, N. E.; Leonard, A. A.; Evans, A. M.; Wasielewski, M. R.; Dichtel, W. R.; Zhang, X.; Chen, L. X.; Schaller, R. D. Photoinduced, Reversible Phase Transitions in All-Inorganic Perovskite Nanocrystals. *Nat. Commun.* **2019**, *10* (1), 504. https://doi.org/10.1038/s41467-019-08362-3.
